# Alteration of synergistic muscle activity following neuromuscular electrical stimulation of one muscle

**DOI:** 10.1002/brb3.87

**Published:** 2012-08-24

**Authors:** Norman Stutzig, Tobias Siebert, Urs Granacher, Reinhard Blickhan

**Affiliations:** Institute of Sportscience, Friedrich-Schiller-UniversitySeidelstraße 20, 07749, Jena, Germany

**Keywords:** Neuromuscular control, NMES, synergistic muscles, triceps surae

## Abstract

The aim of the study was to determine muscle activation of the m. triceps surae during maximal voluntary contractions (MVCs) following neuromuscular electrical stimulation (NMES) of the m. gastrocnemius lateralis (GL). The participants (*n* = 10) performed three MVC during pretest, posttest, and recovery, respectively. Subsequent to the pretest, the GL was stimulated by NMES. During MVC, force and surface electromyography (EMG) of the GL, m. gastrocnemius medialis (GM), and m. soleus (SOL) were measured. NMES of GL induced no significant decline (3%) in force. EMG activity of the GL decreased significantly to 81% (*P* < 0.05), whereas EMG activity of the synergistic SOL increased to 112% (*P* < 0.01). The GM (103%, *P* = 1.00) remained unaltered. Decreased EMG activity in the GL was most likely caused by failure of the electrical propagation at its muscle fiber membrane. The decline of EMG activity in GL was compensated by increased EMG activity of SOL during MVC. It is suggested that these compensatory effects are caused by central contributions induced by NMES.

## Introduction

A multitude of human movements is characterized by different muscles that act together at one joint. Synergists are defined as muscles which actively provide an additive contribution to a particular function during a contraction (Basmajian and Luca 1985). The contributions of muscle activities that act across a joint depend on the direction of contraction and the force (Buchanan et al. 1986; Ashe 1997). Each synergy comprised the coordinated activations of specific muscle groups that included synchronized bursts of electromyografic (EMG) activity for some muscles and asynchronous increases and decreases in EMG activity for other muscles (Enoka 2008). A large number of studies investigated the recruitment patterns of synergistic muscles in different muscle groups. These recruitment patterns are both controlled by the descending drives from supraspinal centers (Ashe 1997) and by the neural circuitry in the spinal cord (Tresch et al. 1999).

A common approach to study control strategies of synergistic muscle activity is to impair one muscle of a muscle group (Sacco et al. 1997; Akima et al. 2002; Kinugasa et al. 2005; de Ruiter et al. 2008). Neuromuscular electrical stimulation (NMES) is an appropriate tool to decrease the activity of one muscle locally (Adams et al. 1993; Vanderthommen et al. 2000). NMES is defined as series of intermittent stimuli to superficial skeletal muscles, with the main objective to trigger visible muscle contractions due to the activation of the intramuscular nerve branches (Maffiuletti 2010).

Akima et al. (2002) examined the muscle activity in the muscle heads of the m. quadriceps femoris at 50% of maximal voluntary contraction (MVC). Between the trials, the m. vastus lateralis was fatigued using NMES for 30 min. It was observed that the muscle activity of the unfatigued muscles was increased compared with the baseline. Thereby, the intended movement task could still be accomplished. These compensatory strategies in muscle synergists were confirmed by de Ruiter et al. (2008). However, the muscle recruitment is circulating until 50% of the MVC and collapse afterward (Allen et al. 2008). Furthermore, at 50% of MVC force can be increased voluntarily. Therefore, Akima et al. (2002) concluded that increased activation of muscle synergists were the result of voluntarily increased central commands.

To avoid the compensation effects which result from muscle recruitment circulation or voluntarily increased central commands, we performed MVC. Moreover, at MVC, the central commands cannot further increase voluntarily (Gandevia 2001), but can be modulated by afferent drives (Mang et al. 2010). It would be interesting if the aforementioned compensatory effects occur at MVC after one muscle within a muscle group is fatigued.

Sacco et al. (1997) fatigued the m. gastrocnemius lateralis (GL) under ischemic conditions using NMES. According to Akima et al. (2002), they found decreased muscle activity in the fatigued GL during MVC. In contrast, the muscle activity of the synergistic m. gastrocnemius medialis (GM) was decreased. The muscle activity of the m. soleus (SOL) was not reported. It was concluded that ischemia lead to inhibitory reflex pathways of the homonymous muscle and lead to inhibition of the unfatigued synergistic muscle. It is unknown if compensatory strategies occur at MVC under nonischemic condition in the triceps surae.

The objective of this study was to examine the compensatory strategies of synergistic muscles during MVC after selective fatigue of one muscle. In contrast to Akima et al. 2002, we choose MVC to avoid voluntarily central contributions to the muscle activation. The GL was selectively fatigued using NMES. Before and after the NMES phase, maximal voluntary isometric plantar flexions were performed. It is hypothesized that the EMG activity of the synergistic SOL and GM are increased after NMES of the GL.

## Methods

Ten healthy men participated in this study (age: 28 ± 4.4 years). All participants were exercise science students involved in different kinds of sports activities (1–2 training sessions/week). The participants were informed about the risks and purposes of the study. Each subject gave written informed consent to participate in the study after experimental procedures were explained. The study was approved by the Institutional Review Board of the Faculty of Social Behavioral Sciences of the Friedrich-Schiller-University Jena, Germany. The study was conducted according to the latest revision of the Declaration of Helsinki.

**Figure 1:**
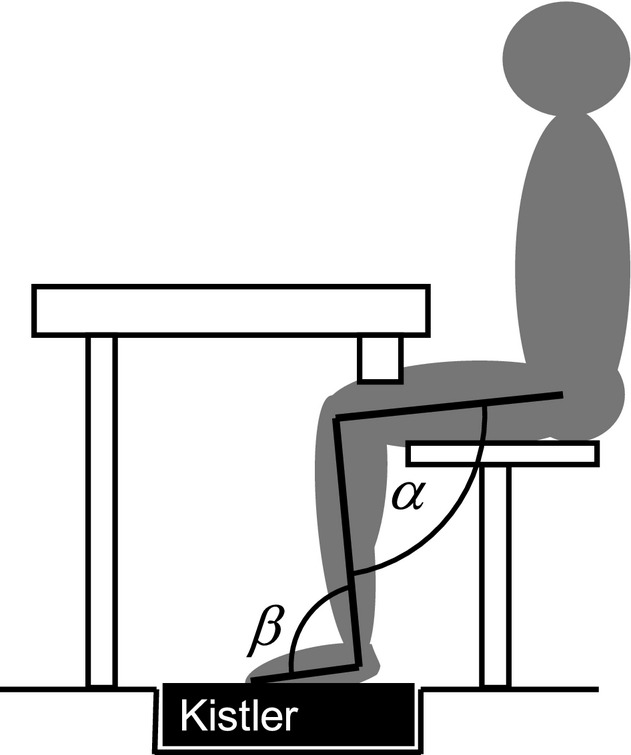
Setup of the participant's position during the measurement. The participant was sitting in a rigid metal frame with one foot on the force plate. The knee and ankle joints were fixed at *α* = 90° and *β* = 90°, respectively.

At least 7 days before the experiment session, participants attended two preparatory sessions to get accustomed to NMES. Participants were placed in a metal frame construction that was specifically built for calf lifts (Fig. 1). The knee joint angle and ankle angle were adjusted at 90°. The dominant foot was placed in the middle of the force plate.

The experimental session began with a standardized warm-up of 3 × 50 skipping and repetitive submaximal plantar flexions. The pretests consisted of three isometric plantar flexed MVCs with the dominant leg separated by a 1-min rest. Right after the last MVC, NMES was started and lasted for 9 min. The posttest comprises three MVC separated by 3 min. To keep up the fatigue in GL, NMES was applied to the GL during the 3-min breaks. Thereafter, participants carried out three MVC at 10, 15, and 20 min during recovery.

During MVC, force was recorded at 1000 Hz using a three-dimensional force plate (Kistler 9281C, Winterthur, Switzerland). Participants were advised to rise up force continuously until a plateau is reached and hold it for 3 sec. To make sure that participants performed a MVC, we considered the methodological recommendations of Gandevia (2001). Briefly, (1) all maximal efforts were accompanied by same instruction and practice; (2) visual feedback was given to the subjects; (3) the investigator gave appropriate and standardized verbal encouragement; (4) subjects were allowed to reject efforts that they did not regard as “maximal.”

For data synchronization purposes, an analog signal from the force platform was used as a trigger and sent to the EMG system.

EMG activities of SOL, GL, and GM were recorded during MVC. We did not measure the antagonistic tibialis anterior, as the activation during MVC is negligible and did not change with fatigue (Patikas et al. 2002). Before the experiment started, the skin was prepared and electrode placements were localized according to the recommendations of SENIAM (Hermens and Roessingh Research and Development BV 1999). Briefly: the skin was dry shaved, abraded, and cleaned with alcohol. Surface EMG activity was detected by two self-adhesive Ag/AgCl^−^ electrodes with a 20 mm interelectrode distance. The signals were preamplified (bandwidth 10–500 Hz) and recorded at 1000 Hz using the Biovision system (Wehrheim, Germany). EMG data were full wave rectified and digitally filtered using a 10 Hz lowpass filter (butterworth, second order) (Arampatzis et al. 2003). The maximal amplitude of the EMG signal was calculated in a time frame of 500 msec around MVC force.

Maximal force and maximal EMG amplitude of SOL, GM, and GL were calculated for nine MVC (three pretest, three posttest, and three recovery). Hence, the three MVC of each condition were averaged.

The GL was fatigued with a portable muscle stimulator (Compex Sport-P, Medicompex SA, Ecublens, Switzerland). Two self-adhesive electrodes (5 × 5 cm) were placed 2 cm proximal to the upper EMG electrode (negative) and 2 cm distal to the lower EMG electrode (positive). Rectangular wave pulse currents (80 Hz) lasting 400 *μ*sec were delivered to the GL. Stimulation intensities were set at the maximal tolerated level and varied between 30 and 50 mA. The on:off ratio was as follows: 6-sec tetanic stimulation followed by a rest of 20 sec, during which the participants were stimulated at 3 Hz.

Data are presented as mean values ± standard error (SE). The data (EMG of SOL, GM, and GL as well as force of MVC) of each test condition (pretest, posttest, recovery) were checked for normal distribution with Kolmogorov–Smirnov test. An analysis of variance (ANOVA) for repeated measures was used to compare dependent variables. The Bonferroni correction was used to analyze differences among pairs of means. To prove the effectiveness of the treatment, the effect sizes (*f*) for ANOVA (for repeated measures) were determined as follows:


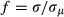


*σ* represents the standard deviation in the population and *σ*_*μ*_ is the standard deviation of the effect (Faul et al. 2007). Furthermore, to determine whether a statistically significant difference is a difference of practical concern, the limits of Cohen (1988) were used: *f*-values <0.2 indicate small, *f*-values <0.5 medium, and *f*-values <0.8 large effects (Cohen 1988). The significance level was set at *P* < 0.05. The Pearson coefficient of correlation was used to examine the relationships between the muscle activities during pretest, posttest, and recovery, respectively. All analyses were performed using Statistical Package for Social Sciences (SPSS, 19.0).

## Results

**Figure 2:**
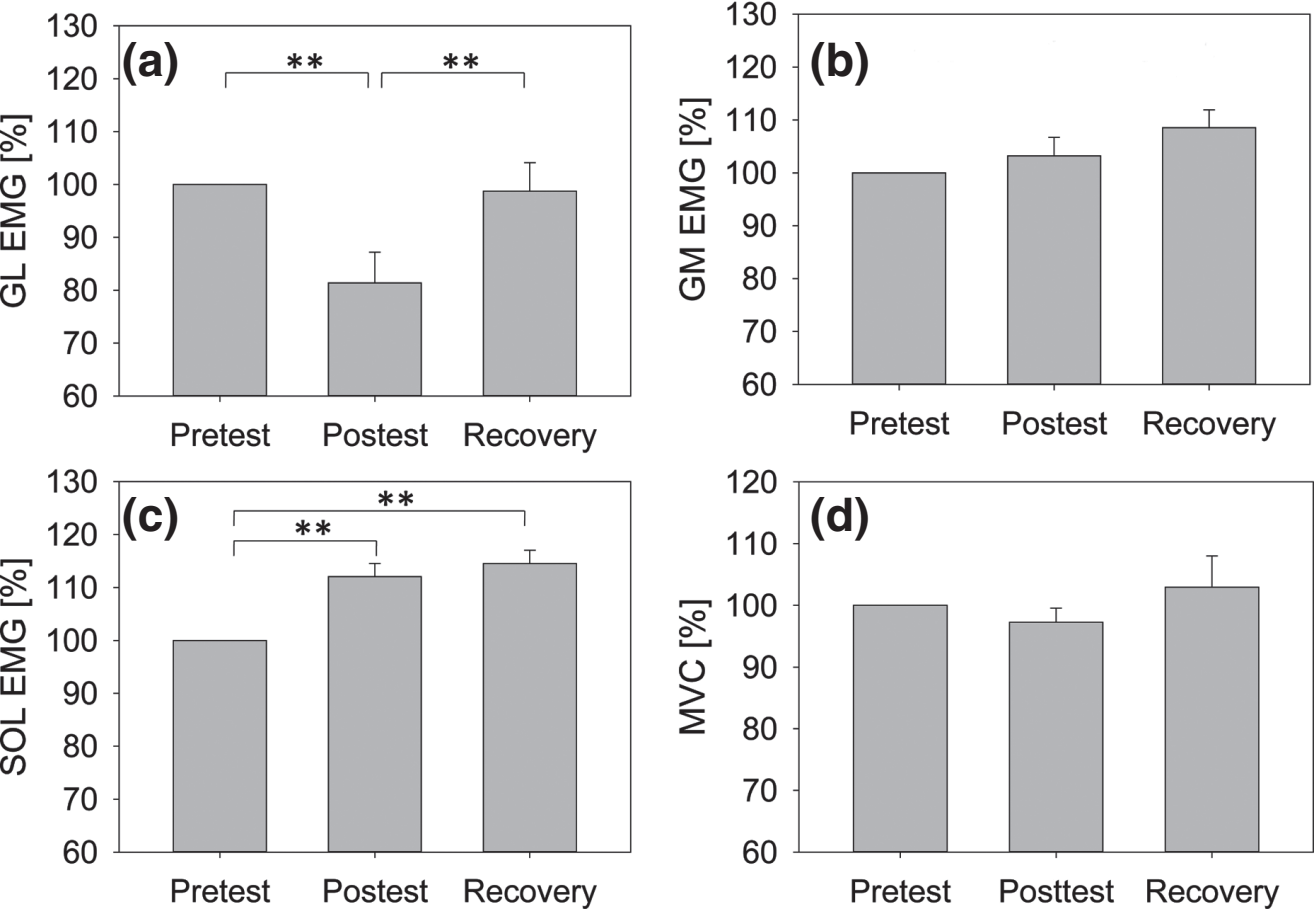
Mean and standard error of the normalized electromyography (EMG) amplitudes of the (A) m. gastrocnemius lateralis, (B) m. gastrocnemius medialis, (C) m. soleus, and (D) force in the pretest, posttest, and recovery phase. The data are normalized to the mean values of the pretest. The statistical analyses were conducted on absolute values. The level of significance was set as follows: *P < 0.05; **P < 0.01.

The force and EMG activity of the muscles are presented in Figure 2. The data in Figure 2 are shown as percentage alteration normalized to the pretest values. EMG activity of the GL significantly decreased during NMES. In the posttest, EMG amplitude decreased from 0.501 ± 0.066 mV to 0.430 ± 0.066 mV (*P* < 0.01, *f* = 0.77, Fig. 2A). During recovery, EMG activity increased to 0.498 ± 0.072 mV (Fig. 2A).

Simultaneously, EMG activity of the SOL increased during NMES from 0.507 ± 0.074 mV to 0.561 ± 0.082 mV. Difference between pretest and posttest turned out to be significant (*P* < 0.01, *f* = 1.18) (Fig. 2C). Furthermore, during recovery, the EMG amplitude still increased up to 0.577 ± 0.085 mV. EMG activity during this phase was significantly higher than during pretest (*P* < 0.01, *f* = 1.18). The results of the GM showed no significant changes between pretest and posttest (Fig. 2B). The EMG amplitude was 0.547 ± 0.076 mV and increased slightly to 0.559 ± 0.076 mV. The difference was not significant. During recovery, the EMG amplitude increased to 0.595 ± 0.084 mV. MVC did not change significantly in posttest as compared to pretest (1062.9 ± 72.4 N vs. 1097.3 ± 76.9 N, respectively). During recovery, force values increased to 1111.9 ± 66.0 N (Fig. 2D).

We calculated correlations between muscle activities during pretest and during posttest, respectively. The results showed high correlations between SOL and GL in pretest (*r* = 0.803, *P* < 0.01) and posttest (*r* = 0.770, *P* < 0.01). The GL and GM were highly correlated in pretest (*r* = 0.818, *P* < 0.01) and posttest (*r* = 0.847, *P* < 0.01). Furthermore, there were medium correlations between GM and SOL in pretest (*r* = 0.671, *P* < 0.05) and no significant correlations in posttest (*r* = 0.595, *P* > 0.05).

**Figure 3:**
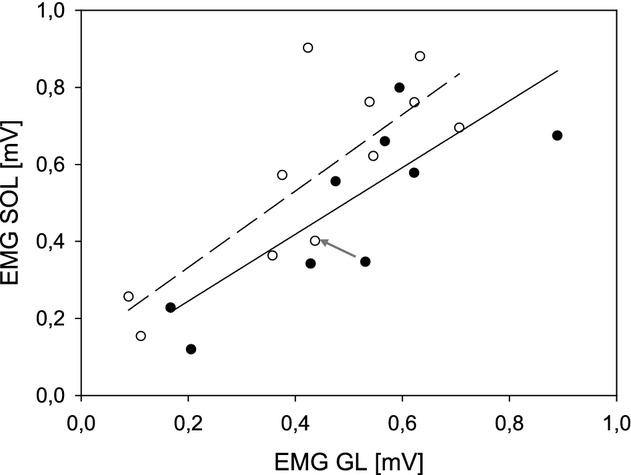
Correlations of the electromyography (EMG) activity of m. gastrocnemius lateralis (GL) and m. soleus (SOL) in pretest (open circles) and posttest (filled circles), respectively. The regression lines are presented for EMG activity of GL and SOL in pretest (solid line) and posttest (dashed line). The gray arrow symbolized for one participant that the regression line shifted to the left upper side during posttest.

Comparing the regression lines of SOL pretest versus GL pretest with SOL posttest versus GL posttest, one can see a shift to the left upper side. This can be ascribed to the decrease in GL activity and the increase in SOL activity as indicated exemplarily by one participant (Fig. 3, gray arrow).

## Discussion

This is the first study that demonstrated an increase in synergistic EMG activity during maximal voluntary isometric contractions following NMES of one muscle. Due to the NMES of the GL, the EMG activity decreased to 81% in posttest. This decline was compensated by the EMG activity of SOL that increased to 112% in posttest. The force during MVC did not change significantly after NMES of the GL (Fig. 2D).

Following sustained NMES of the GL, voluntary GL muscle activity during maximal isometric contractions was reduced (Fig. 2A). The results are in line with other studies that found decreased EMG amplitudes after high-frequency NMES (Boerio et al. 2005). The decline in EMG activity occurs due to failure of electrical propagation at the muscle fiber membrane of the GL induced by high-frequency fatigue (Cairns and Dulhunty 1995; Badier et al. 1999). Furthermore, studies using the interpolation twitch technique showed that electrical stimulation of the triceps surae lead to central fatigue (Boerio et al. 2005) accompanied by a force decline. In our studies, the force did not change significantly (*P* = 0.388) after NMES of the GL. For that reason, we assume that the decline in GL in the presented study occurred more prominently due to peripheral fatigue than due to central fatigue (Badier et al. 1999).

Reducing knee angle leads to reduced GM length and decreased muscle activation during MVC (Cresswell et al. 1995; Arampatzis et al. 2006). This may be due to (1) the “drive,” i.e., the neural outflow from spinal motor neurons, may be reduced to a shortened muscle; (2) neuromuscular transmission–propagation in a shortened muscle may be impaired, and (3) shortening a muscle may alter the electrode configuration with respect to the recording volume, thereby resulting in less myoelectric activity recorded (Cresswell et al. 1995).

In our experiments, knee and ankle angle and therefore the muscle length were kept constant during pre- and posttests. However, tendon length may increase during repeated high efforts (Kubo et al. 2001). This would lead to shorter fascicles and therefore decreased muscle activations in all muscles of the m. triceps surae. However, we found only decreased muscle activation in GL but not in GM and even increased muscle activation in SOL.

As the GL activity decreased due to fatigue, compensatory strategies exist to produce the same force during the MVC. Those strategies were found in higher activation of synergistic muscle SOL in particular. Akima et al. (2002) found increased activations of the m. rectus femoris, m. vastus medialis, and m. vastus intermedius in voluntary dynamic knee extension at 50% of MVC after NMES of the m. vastus lateralis. Similarly, our results show increased activity in the SOL (Fig. 2C). Contrary to Akima et al. (2002), we used maximal isometric plantar flexions and examined the muscle activity in SOL, GM, and GL after NMES of GL.

There are several reasons why activity of synergistic unfatigued muscles is increased due to selective fatigue of the GL. It is possible that increased afferent drive increase the central activation at least in the SOL and GM. It is known that NMES, as we used in our experiment, provokes spinal contributions via afferent drives (Gondin et al. 2006) and increases the excitability of spinal reflexes (Trimble and Harp 1998; Kitago et al. 2004). The increased spinal reflex excitability can be sustained for 16 min (Kitago et al. 2004) after stimulation. Furthermore, Ia fibers of the GL are cross-linked to the α motoneurons of the GM and SOL (Nichols 1999). From animal experiments, it is known that spinal cross-linkages between GL and SOL are strong, while they are weaker between GL and GM (Eccles et al. 1957; Nichols 1989). The muscle spindles are activated during MVC (Hagbarth et al. 1986) and contribute to the voluntary force production up to 30% (Gandevia et al. 1990). Considering the aforementioned facts, it is possible that in our experiments the NMES of the GL provoked a latent higher excitability of the spinal reflexes. The consequence would be higher muscle activity in SOL and comparably low increase in GM muscle activation during MVC. This explanation is based on several assumptions, which we did not measure directly. Therefore, it is hypothetical and needs to be proved in further studies.

NMES also affects supraspinal areas (Maffiuletti 2010). Using magnetic resonance imaging Smith et al. (2003) found, the higher the current intensity the greater the response of different brain areas. Furthermore, Mang et al. (2010) stimulated the peroneal nerve with transcranial magnetic stimulation at different frequencies (20, 50, 100, 200 Hz) and measured increased corticospinal excitability. Corticospinal pathways were solely increased after high-frequency stimulation at 50 and 100 Hz and lasted for 24 min. This increased corticospinal excitability may also contribute to the EMG activity in the m. triceps surae.

At the peripheral level, increased EMG activity can result from decrease in muscle fiber conduction velocity (Rongen et al. 2002). Rongen et al. (2002) showed that the EMG amplitude increased whereas the muscle fiber conduction velocity decreased during sustained isometric contractions under ischemic conditions. During high-frequency NMES, muscle metabolism is highly utilized (Shenton et al. 1986) and the muscle pH decreased (Vanderthommen et al. 2003). For that reason, it is possible that changes of the muscle fiber conduction velocity can occur, but only in the stimulated GL. That does not explain the increased activity in SOL.

Our results indicated different activation strategies in synergistic muscles (SOL, GM). According to the results of Akima et al. (2002) and de Ruiter et al. (2008), it was hypothesized that EMG activity of both synergistic muscles would increase. On the other hand, Sacco et al. (1997) reported decreased EMG activity in GM after NMES of GL. However, this decline in EMG activity is attributed to the ischemia conditions in their study. In our study, it is assumed that EMG activity of GM was affected by the NMES of the neighboring GL (Adams et al. 1993). This might induce an unaltered EMG activity in GM (*P* = 1.00) during NMES. Furthermore, in recovery, EMG activity of GM increased slightly compared with the baseline. The high correlations (*r* = 0.847, *P* < 0.01) between GM and GL during recovery support this assumption.

During recovery, the activation of the GL goes back to baseline values. The muscle activation of GM is unaltered and muscle activation of SOL is still increased. Therefore, one would expect significant increase of force. In fact, force does not increase significantly. This might be due to metabolic fatigue in the stimulated GL (Shenton et al. 1986; Vanderthommen et al. 2003).

In fact, EMG activity in the SOL increased after NMES of the GL at high frequencies, but not EMG activity of GM. Further studies are needed to clarify whether EMG activity of the synergistic muscles results from peripheral changes or improved central activations.

In conclusion, a progressive fatigue protocol of the GL by means of NMES resulted in (a) unaltered force during maximal voluntary isometric plantar flexions, (b) increased synergistic muscle activity of the SOL. It is suggested that these compensatory effects are caused by central contributions induced by NMES. The results provide new insights in neuromuscular control of synergist muscles.
